# Maleic acid as a root canal irrigant- a scoping review

**DOI:** 10.1186/s12903-025-07361-9

**Published:** 2025-12-08

**Authors:** Tanya Kapur, Edlin Glane Mathias, Sonia Gupta, Arathi P. Rao, Nidambur Vasudev Ballal

**Affiliations:** 1 Department of Conservative Dentistry and Endodontics, Manipal College of Dental Sciences, Manipal, Manipal Academy of Higher Education, Manipal, Karnataka India; 2 Centre for Evidence-informed Decision-making, Department of Health Technology and Informatics, Prasanna School of Public Health, Manipal Academy of Higher Education, Manipal, Karnataka India; 3 Dept of Global Public Health Policy and Governance, Prasanna School of Public Health, Manipal Academy of Higher Education, Manipal, Karnataka India; 4 Department of Conservative Dentistry and Endodontics, Manipal College of Dental Sciences, Manipal, Manipal Academy of Higher Education, Manipal, Karnataka India

**Keywords:** Chelating agents, Endodontics, Maleic acid, Root canal irrigants, Smear layer

## Abstract

**Background:**

Root canal treatment (RCT) requires thorough removal of the smear layer to ensure proper sealing, reduce microbial contamination, and enhance treatment success. Chelating agents such as ethylenediaminetetraacetic acid (EDTA) are widely used, but they show limitations, especially in the apical third. Maleic acid (MA), a mild organic acid with low pH, has been proposed as a promising alternative due to its superior demineralising and adhesive-enhancing properties.

**Aim:**

To evaluate the role of MA in root canal therapy, with emphasis on smear layer removal, antimicrobial efficacy, dentin-related effects, and interactions with dental materials.

**Methods:**

The review followed PRISMA-ScR 2018 guidelines and Arksey and O’Malley’s six-stage framework. A comprehensive search of PubMed, Scopus, Embase, and Web of Science was conducted up to October 2024. Study eligibility was defined using the PICO framework: (P) human teeth, dentin slices, bacterial cultures, and dental material specimens studied; (I) maleic acid as a root canal irrigant; (C) comparisons with EDTA, sodium hypochlorite, citric acid, and newer chelators; (O) outcomes including smear layer removal, growth factor release, effects on dental materials, antimicrobial efficacy, dentin mineral content, physical and mechanical properties, and bond strength. 71 studies were included.

**Results:**

7% MA was highly effective in smear layer removal, particularly in the apical third, surpassing 17% EDTA and other irrigants. MA enhanced dentin wettability, sealer penetration, and push-out bond strength, and showed superior efficacy in removing calcium hydroxide and intracanal medicaments. It also promoted growth factor release, supporting regenerative potential. While antimicrobial activity was moderate alone, it improved significantly when combined with chlorhexidine or cetrimide. However, MA reduced dentin microhardness and mineral content and negatively impacted calcium silicate–based materials such as mineral trioxide aggregate and Biodentine.

**Conclusion:**

7% MA is a potent root canal irrigant with consistent superiority in apical smear layer removal and unique advantages in adhesion and regeneration. Its demineralising effects highlight the need for controlled application, with shorter conditioning times (1–3 min) optimising outcomes while minimising risks. Overall, MA represents a promising, less cytotoxic alternative to EDTA, but standardised clinical protocols and rigorous in vivo validation are essential for its safe integration into endodontic practice.

**Supplementary Information:**

The online version contains supplementary material available at 10.1186/s12903-025-07361-9.

## Introduction

Root canal treatment (RCT) involves the cleaning and shaping of the root canal system, subsequently filling the pulpal space with a sterile material, with the objective of restoring and preserving a tooth that is significantly damaged or infected. The intricate shape of root canals complicates the disinfection of the pulp cavity, potentially affecting the efficacy of RCT. An effective endodontic procedure requires specialised instruments and irrigating solutions to thoroughly clean and shape the root canals. Root canal instrumentation produces a layer of organic and inorganic substances referred to as the smear layer [1]. In 1975, McComb and Smith [2] initially documented the existence of this layer in instrumented root canals. The smear layer consists of irregular amorphous deposits of inorganic and organic dentin debris, encompassing pulpal tissue, necrotic odontoblastic processes, bacteria, and their metabolic byproducts.

Thorough removal of the smear layer from the root dentin is essential for successful endodontic therapy [3]. The smear layer has been demonstrated to harbor and safeguard bacteria within the dentinal tubules [4]. The smear layer is a poorly adhering, heterogeneous structure that can be readily dislodged from the underlying dentin, potentially leading to bacterial contamination and leakage between the filling material and the dentinal walls [2–5]. It may also obstruct the infiltration of the irrigating fluid and intracanal medicines into the tubules [6].

Gençoǧlu et al. [7] indicated that the smear layer diminishes the capacity of gutta-percha to adhere adequately to the canal walls, irrespective of the condensation procedure employed. Additionally, the smear layer serves as a barrier between the canal wall and root filling materials, thereby undermining the establishment of an adequate seal [8]. Pashley et al. [9] conducted a study that revealed the presence of microchannels, measuring between 1 and 10 μm in thickness, between the root filling material and the dentinal wall in the presence of a smear layer.

The outcome of RCT may be influenced by the adverse effects of these channels on the apical and coronal seals, as well as their capacity to dissolve and create voids in insufficiently filled root canals [10]. Consequently, the total removal of the smear layer is essential for the efficacy of root canal therapy.

Chelating agents are used together with irrigating solutions during biomechanical preparation for various purposes, such as the removal of hard tissue debris and the inorganic component of the smear layer, facilitating instrumentation in calcified canals, and conditioning dentinal walls to improve the adhesion of filling materials [11]. Chelating agents interact with the calcium ions in the tooth structure, leading to the decalcification of dentin. The efficacy of chelating agents in decalcification is significantly affected by variables like application duration, solution pH, and concentration levels [12]. In endodontics, many chelating chemicals and acids have been utilised to remove the smear layer. Ethylenediaminetetraacetic acid (EDTA) is a widely utilised chelating solution in endodontics. It aids in the elimination of the smear layer and conditions the dentin walls for enhanced adherence with the dental material [13]. The strategy for efficiently eliminating both organic and inorganic constituents of the smear layer includes the application of sodium hypochlorite (NaOCl) succeeded by EDTA or citric acid (CA). EDTA possesses several drawbacks, including cytotoxicity, diminished adhesion of resin sealers, decreased efficacy in smear layer removal in the apical third, loss of available chlorine when combined with NaOCl, precipitate formation with chlorhexidine (CHX), and ineffectiveness in removing calcium hydroxide from the root canal when utilised independently [14]– [15].

Although CA is a weak acid, its antibacterial efficacy and reduced toxicity have been shown to be comparable to those of EDTA [16]. It has demonstrated superior advantages in regenerative endodontic operations by facilitating increased TGF-β1 release relative to traditional 17% EDTA. Nevertheless, its application may diminish dentin microhardness and provoke decalcification and erosion, especially when utilised prior to NaOCl. Currently, the information regarding the impact of CA on sealer adherence and its adaptation to root canal walls is contradictory, attributed to inconsistency among studies [17].

Maleic acid (MA) has emerged as a potential irrigant due to its effective smear layer removal in the apical third, reduced cytotoxicity, and capacity to promote sealer adherence by improving dentin surface roughness and wettability [14, 18]. Numerous investigations have indicated that MA exhibits enhanced efficacy in the elimination of calcium hydroxide relative to EDTA and citric acid [19, 20]. Nevertheless, the evidence remains fragmented across separate in vitro studies, and concerns continue over its capacity for dentin demineralisation and interactions with calcium silicate–based substances. Consequently, a thorough mapping of the existing research is essential to elucidate the comprehensive advantages, drawbacks, and clinical implications of MA as a root canal irrigant. This scoping review was thus undertaken to consolidate current knowledge, identify gaps, and provide direction for future research.

## Methods

Utilising a scoping review methodology, we analysed the current literature to identify research gaps and enhance our comprehension of the primary outcomes associated with the use of MA as a root canal irrigant and its effects. The protocol for this scoping review was not registered in PROSPERO, as PROSPERO does not currently accept scoping review protocols. This scoping review was carried out in compliance with the “Preferred Reporting Items for Systematic Reviews and Meta-Analyses (PRISMA) Extension for Scoping Reviews Checklist 2018.” [21] We adhered to the six-stage methodological framework for scoping reviews established by Arksey and O’Malley [22], which comprises the following steps:

### Step 1: specify the research question

- ☐ What is the effect of MA on smear layer removal?
- ☐ What are the other effects of MA when used as an irrigant, in root canal treatment?

The Population, Intervention, Comparison, Outcome(s) and Criteria for study design to identify the studies was used.

#### Population

Human teeth, dentin slices, material specimens, bacterial cultures.

#### Intervention

MA as an irrigation solution in RCT.

#### Comparison

Other root canal irrigants (e.g., EDTA, NaOCl, CA).

#### Outcomes

Effects of MA, including smear layer removal, effect on growth factors release, effects on dental materials, antimicrobial efficacy, effect on inorganic mineral contents of dentin, effect on physical and mechanical properties of root canal dentin and effect on bond strength of post to root dentin.

## Study designs

Only in-vitro and ex-vivo studies published in English were included to ensure precise interpretation of methods and findings, as translating non-English papers may lead to inaccuracies or inconsistencies. Protocols, editorials, and reviews were excluded due to their insufficient data for information synthesis. This review excluded grey literature to maintain methodological rigour, as grey literature frequently lacks standardised peer review and comprehensive reporting, potentially undermining the reliability and reproducibility of findings.

### Step 2: identify the relevant literature

A comprehensive search technique was developed by detecting keywords from the Medical Subject Headings (MeSH) browser [23]. The research utilised the phrases “maleic acid,” “root canal irrigants,” “smear layer,” “root canal,” “chelating agents,” “endodontics,” and “dental.” Until October 10, 2024, four databases—PubMed (MEDLINE), Scopus (Elsevier), Embase (Elsevier), and Web of Science (Clarivate)—were searched electronically. Search queries from PubMed (NCBI) were converted across many databases with a polyglot search translation software [24]. The Boolean operators “AND” and “OR” were employed to combine the search. This review considered all published original research studies without constraints on publication date. The search strategy for all databases is detailed in Appendix 1.

### Step 3: selection of studies

The Rayyan software facilitated the importation of database search results [25]. Title and abstract screening was conducted independently by two review authors (T.K. and N.V.B.), with any discrepancies resolved through discussion. Full text screening was conducted separately by two reviewers, T.K. and N.V.B., with discrepancies resolved through consultation with a third reviewer, E.G.M. Studies without full text or necessary information were eliminated following diligent efforts to get the material, including reaching out to the study authors when possible.

### Step 4: charting the data

Data extraction was conducted independently by two reviewers (T.K. and N.V.B.) utilising a pre-piloted Microsoft Excel spreadsheet. The extracted variables comprised the author’s name, publication year, study sources, study characteristics, and assessed outcomes, including smear layer removal, antimicrobial activity, growth factor release, effects on mineral content, properties of dentin, and bond strength. Discrepancies were addressed via discussion or consultation with a third reviewer (E.G.M.).

Studies with incomplete or ambiguous data were addressed systematically. In cases where the full text was not accessible, efforts were made to obtain it via institutional subscriptions or by reaching out to the authors. The study was excluded from the final synthesis when essential methodological or outcome data were unavailable, thereby ensuring accuracy and transparency. The final data set was structured narratively and summarised in tables to convey study characteristics and key findings.

### Step 5: collecting, summarizing, and reporting results

Results were compiled through a narrative approacffh, supplemented by tables and figures. This study outlines the characteristics of the settings and compares the effects of MA with other irrigants, along with the results obtained.

### Step 6: stakeholder consultation

We were unable to hold stakeholder consultations because of time and financial limitations.

## Results

### Study selection process

Electronic searches were conducted on PubMed (MEDLINE), Scopus (Elsevier), Web of Science (Clarivate), and EMBASE (Elsevier). Rayyan software, utilised for reference management, duplicate removal, and screening facilitation, was employed to eliminate duplicates. Following the full-text and title-abstract screenings, 71 articles were included for analysis. The remaining articles were excluded for the following reasons: utilisation of alternative irrigants (*n* = 1), failure to assess the effect of MA (*n* = 2), or lack of a definitive effect of MA (*n* = 1).

**Fig. 1:**
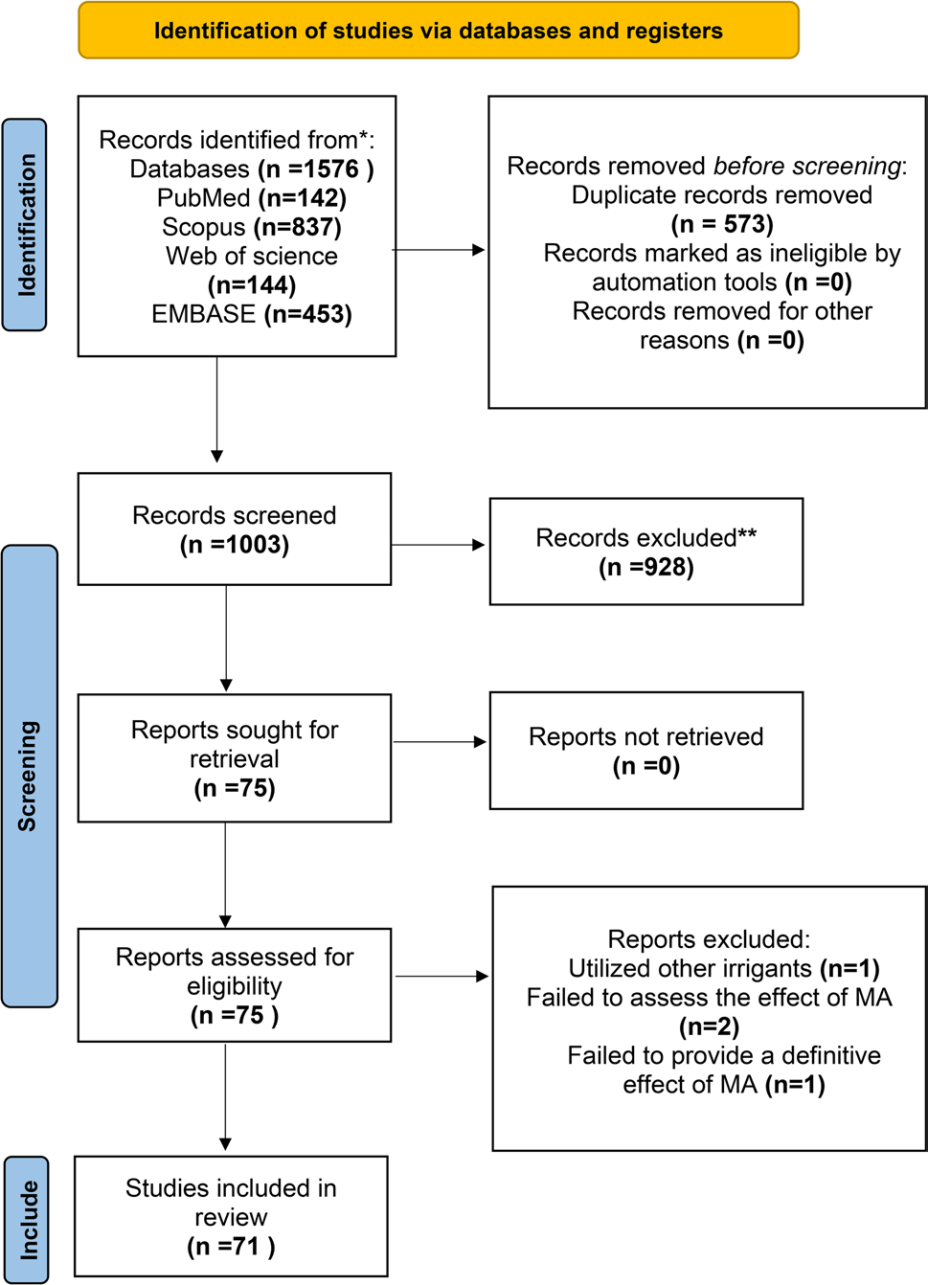
Study selection process

The PRISMA 2020 flow diagram for study selection process is shown in Fig. 1.

### Characteristics of included studies

#### Study settings

The studies included were conducted in various nations. Forty-six investigations were undertaken in India [13–15, 18–20, 26–66], thirteen studies in Turkey [67–78], one research each in Italy [79], Saudi Arabia [80], the Republic of Cyprus [81], and Brazil [82], two studies in China [83, 84], and six in Spain [85–90].


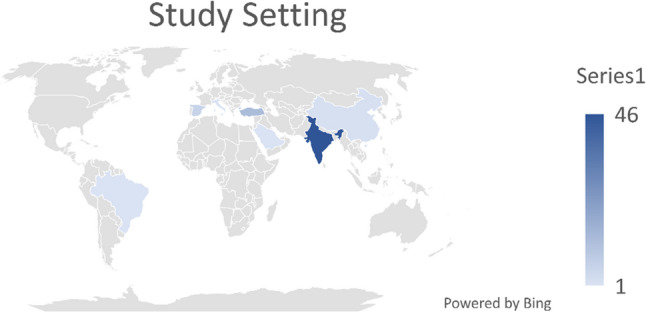


#### Study characteristics

The characteristics of the included studies are outlined in Table 1 for the smear layer and Table 2 for additional effects.

**Table 1:** Effect on smear layer

Author Year Study source	Aim	Sample size and groups	Results
Attur et al. 2016 [13] Journal of International Society of Preventive and Community Dentistry	To evaluate the efficiency of different endodontic irrigants in the removal of smear layer through scanning electron microscopic image analysis.	45 single-rooted mandibular premolar teeth Group 1:: 17% EDTA Group 2:: 7% MA, Group 3:: 2% CHX	17% EDTA outperformed 7% MA and 2% CHX in smear layer removal across the coronal, middle, and apical thirds of the root canal. The most effective removal was seen with 17% EDTA, while 2% CHX was the least effective.
Ballal et al. 2009 [15] Journal of Endodontics	To assess, by SEM analysis, the ability of 17% EDTA and 7% MA in the removal of the smear layer from the human root canal system	80 single-rooted human anterior teeth Group 1: 17% EDTA + 2.5% NaOCl Group 2: 7% MA + 2.5% NaOCl Group 3: 0.9% saline	In the coronal and middle thirds, 17% EDTA and 7% MA were equally effective in smear layer removal. In the apical third, 7% MA was significantly more effective than 14% EDTA.
Kuruvilla et al. 2015 [19] Journal of Conservative Dentistry	The purpose of this study is to evaluate and compare the efficacy of 17% EDTA, 18% etidronic acid, and 7% MA in smear layer removal using SEM image analysis.	30 mandibular premolars Group 1:17% EDTA Group 2: 18% etidronic acid Group 3: 7% MA	The three irrigants removed the smear layer at different levels, with 7% MA being more effective than 17% EDTA and 18% etidronic acid in the apical third of the root canal.
Ballal et al. 2019 [27] Acta Odontologica Scandinavica	To evaluate SmearOFF, 7% MA and two different preparations of EDTA in smear layer removal.	50 single-rooted teeth Group 1: SmearOFF Group 2: 7% MA Group 3: 18% EDTA (pH 11.4) Group 4: 17% EDTA (pH 8.5) Group 5: 0.9% saline	SmearOFF and 17% EDTA (pH 8.5) effectively removed the smear layer in the coronal and middle thirds, while 7% MA was superior in the apical third. 18% EDTA (pH 11.4) and saline showed poor performance.
Ballal et al. 2010 [28] International Journal of Clinical Dentistry	The smear layer removing ability of different concentrations (3%, 5% or 7%) of MA and a commonly used EDTA based paste preparation (RC-prep) both followed by irrigation with 2.5% NaOCl on instrumented root canal surface	50 maxillary anterior teeth Group 1: 3% MA followed by 2.5% NaOcl Group 2: 5% MA followed by 2.5% NaOcl Group 3: 7% MA followed by 2.5% NaOcl Group 4: RC prep followed by 2.5% NaOcl & final irrigation 17% EDTA Group 5: 0.9% saline	The three MA concentrations were more effective than 17% EDTA (RC-prep) in removing the smear layer from both the middle and apical thirds of the root canal.
Ballal et al. 2016 [29] Journal of Dentistry	To evaluate the canal wall smear layer removal capacity and mineral content distribution of root canal dentine after irrigation with QMix, 7% MA and 17% EDTA.	40 single-rooted teeth Group 1: 7% MA + 2.5% NaOCl Group 2: 17% EDTA + 2.5% NaOCl Group 3: QMix + 2.5% NaOCl Group 4: 0.9% saline	7% MA outperformed QMix and 17% EDTA in smear layer removal in the apical third. QMix reduced calcium more, while MA and QMix lowered phosphorus and magnesium. EDTA was more effective at reducing carbon, and MA significantly reduced oxygen.
Saju et al. 2024 [30] Endodontology	To assess the effect of XP-Endo Finisher (XPF) in conjunction with three different chelating agents, namely, 0.2% CNP, 17% EDTA and 7% MA on residual debris and smear layer on the root canal walls of the mandibular premolars.	80 mandibular premolars Group 1: XPF + 0.2% CNP Group 2: XPF + 17% EDTA Group 3: XPF + 7% MA Group 4: XPF + 2.5% NaOCl	XPF + 0.2% CNP was the most effective in removing the smear layer across all root canal sections, followed by XPF + 17% EDTA and XPF + 7% MA. Smear layer removal was least effective in the apical third for all groups.
Mullick et al. 2023 [31] Pesquisa Brasileira em Odontopediatria e Clínica Integrada	To assess the efficacy of 5% GA, 17% EDTA and 7% MA, in removing the smear layer	40 single-rooted human teeth Group 1: 05% GA Group 2: 17% EDTA Group 3: 7% MA Group 4: Distilled water	7% MA as a final irrigating solution is more efficacious than 17% EDTA and 5% GA in eliminating the smear layer from the apical portion of the root canal.
Mankeliya et al. 2021 [32] Journal of Contemporary Dental Practice	To evaluate and compare the efficacy of 17% EDTA, 18% etidronic acid, 10% CA, and 7% MA in the removal of smear layer at the apical third of the root canals.	60 single-rooted teeth Group 1: 17% EDTA Group 2: 18% etidronic acid Group 3: 10% citric acid Group 4: 7% MA	7% MA demonstrated superior smear layer removal in the apical third compared to all other groups. 10% CA was more efficient than EDTA and etidronic acid. With no significant difference between all the study groups, except MA.
Gupta et al. 2018 [33] Journal of Dentistry	To compare efficacy of various irrigating solutions for smear layer removal and dentin microhardness.	50 single-rooted teeth Group 1: 17% EDTA Group 2: 7% MA Group 3: 10% MA Group 4: MTAD Group 5: saline	7% MA was as effective as MTAD
Butala et al. 2017 [34] Saudi Endodontic Journal	To assess the ability of 7% MA, 0.5% PAA, and 17% EDTA in removing smear layer from root canal system of human teeth using SEM.	35 anterior teeth with single roots Group 1: 7% MA Group 2: 0.5% PAA Group 3: 17% EDTA Group 4: 0.9% saline	In the coronal thirds of the root canal, no significant difference was found between 17% EDTA and 7% MA in smear layer removal. However, 7% MA was significantly more effective than 0.5% PAA and 17% EDTA in removing the smear layer from the middle and apical thirds of the root canal system.
Kaushal et al. 2020 [35] Journal of Conservative Dentistry	To determine which irrigant effectively removes the smear layer from the coronal, middle, and apical third of the root canal.	120 single-rooted mandibular premolars Group 1: 17% EDTA Group 2: 10% CA Group 3: 7% MA Group 4: saline	7% MA and 10% CA were equally effective in removing the smear layer from the coronal and middle thirds, but 7% MA performed better in the apical third. When comparing CA and EDTA, both were equally effective in the coronal and middle thirds, but 10% CA outperformed 17% EDTA in the apical third.
Nabi et al. 2020 [36] Endodontology	To evaluate the effect of diode laser with MA and EDTA on smear layer removal from root canals.	160 mandibular premolars Group 1: 17%EDTA; Group 2: diode + 17%EDTA Group 3: 7%MA Group 4: diode + 7MA	The diode laser combined with 7% MA was significantly more effective than 17% EDTA.
Meshram et al. 2016 [37] Contemporary Clinical Dentistry	To compare the effectiveness of EDTA, MA, and DMSA against the combination of these with NaOCl in the removal of smear layer.	140 anterior teeth Group 1: normal saline, Group 2: 17% EDTA, Group 3: 17%EDTA + NaOCl Group 4: 7% MA, Group 5: 7% MA + NaOCl, Group 6: 10% DMSA, Group 7: 10% DMSA + NaOCl	10% DMSA + NaOCl was significantly more effective than all other groups in both the middle and apical thirds, with better performance in the middle third compared to the apical third.
Rao et al. 2021 [38] Saudi Endodontic Journal	To assess the effect of SmearOFF, 7% MA and 17% EDTA when combined with NaOCl in removal of smear layer from curved root canals.	40 mandibular molars Group 1: SmearOFF, Group 2: 7% MA, Group 3: 17% EDTA, Group 4: 0.9% saline	No significant difference between the SmearOFF and 7% MA groups in the coronal, middle, and apical thirds. In the 17% EDTA group, smear layer removal was similar in the coronal and middle thirds, but less effective in the apical third compared to SmearOFF and 7% MA. Overall, while all irrigants worked well in the coronal and middle thirds, SmearOFF and 7% MA were notably more effective than 17% EDTA in the apical third.
Varghese et al. 2017 [39] Saudi Endodontic Journal	This study evaluates the demineralization effect of a chelating agent, MA when used as a root biomodifier on the cemental surface.	30 single-rooted anterior teeth Group 1: 50% CA Group 2: 17% EDTA Group 3: 7% MA Group 4: saline	All three test agents effectively eliminated the smear layer at both time intervals. Intergroup comparison showed 7% MA to have better smear layer removal ability than 50% CA and 17% EDTA.
Ulusoy et al. 2013 [67] Australian Endodontic Journal	To compare the effects of different irrigants on root dentine microhardness, erosion and smear layer removal.	72 root dentine slices Group 1: 17% EDTA + 2.5% NaOCl Group 2: 7% MA + 2.5% NaOCl Group 3: 1.3% NaOCl + MTAD Group 4: Smear Clear + 2.5% NaOCl Group 5: 5% NaOCl, Group 6: saline	7% MA with a 5-minute application caused the greatest reduction in root dentine microhardness, followed by 17% EDTA and MTAD. Smear Clear, 17% EDTA, MTAD, 7% MA effectively removed the smear layer in the coronal and middle thirds, while 7% MA produced much cleaner dentinal tubules in the apical third compared to the other solutions.

*MA* Maleic acid, *EDTA* ethylenediaminetetraacetic acid, *NaOCl* sodium hypochlorite, *CNP* chitosan nanoparticles, *GA* glycolic acid, *PAA* peracetic acid, *CHX* chlorhexidine, *CA* citric acid, *SEM* scanning electron microscope, *DMSA* dimercaptosuccinic acid, *MTAD* Mixture of Tetracycline, Acid, Detergent

**Table 2:** Additional effects

Author Year Study source	Aim	Sample size and groups	Results
Ballal et al. 2009 [15] Oral surgery, Oral Medicine, Oral Pathology, Oral Radiology, and Endodontics	To compare aqueous solutions of EDTA with that of MA for their cytotoxic effect on Chinese hamster fibroblasts (V79) cells growing in vitro	Exponentially grown V79 cells EDTA (0.05% to 1.0%) or MA (0.05% to 1.0%)	Clearly demonstrated the significantly less toxic effect of 7% MA at a comparable dose of 14% EDTA
Neelakantan et al. 2011 [16] International Endodontic Journal	To investigate the impact of dentine conditioning on sealing ability and dentine bond strength of an epoxy resin sealer.	90 single-rooted teeth Group 1: water Group 2: 3% NaOCl Group 3: 17% EDTA Group 4: 7% MA or 2% CHX	Leakage decreased over time, with POBS highest in the coronal and lowest in the apical thirds. Final application of a decalcifying agent reduced leakage and increased POBS, unlike in groups where NaOCl was applied last, which negated the decalcifying agents effect.
Pallepagu et al. 2022 [20] Cureus Journal of Medical Science	To evaluate the efficacy of 10% MA in comparison with 17% EDTA in the removal of intracanal medicaments from the root canal system.	48 single-rooted mandibular premolars Group 1: Metapex, Group 2: Odontopaste, subgroup: 17% EDTA, 10% MA 0.9% saline	Both 17% EDTA and 10% MA removed Odontopaste significantly better than Metapex, with 17% EDTA being more effective. However, 10% MA was more effective than 17% EDTA in removing Metapex.
Ballal et al. 2022 [40] Journal of Endodontics	To analyze the effect of 7% MA on root conditioning of an infected root canal on the release of TGF-β1.	Single-rooted human teeth Group 1: 1.5% NaOCl Group 2: 7% MA Group 3: 17% EDTA, Group 4: combination of 1.5% NaOCl with 17% EDTA or 7% MA	7% MA with 1.5% NaOCl released more growth factors than 17% EDTA with 1.5% NaOCl in both biofilm and nonbiofilm groups. Nonbiofilm samples treated with 7% MA alone showed higher growth factor release than those treated with 17% EDTA, but no significant difference was found in the biofilm samples between the two treatments.
Ballal et al. 2019 [41] Microscopy Research and Technique	To evaluate the efficacy of different irrigation protocols in removing two tricalcium silicate-based sealers from simulated root canal irregularities and root canal walls	140 single-rooted teeth Group 1: MTA Fillapex Group 2: BioRoot RCS Subgroups: 2.5% NaOCl-17% EDTA, 5% NaOCl/9% DualRinse HEDP, 2.5% NaOCl-7% MA, 2.5% NaOCl-17% EDTA (Er: YAG LAI), 2.5% NaOCl/9% DualRinse HEDP (LAI), 2.5% NaOCl-7% MA (LAI), Distilled water	MA and DualRinse HEDP removed more MTA Fillapex from the grooves than EDTA, regardless of the activation method used.
Ballal et al. 2017 [42] Journal of Dentistry	To compare the effect of QMix, 7% MA, and 17% EDTA on the microhardness, flexural strength and microstructure of MTA; ProRoot MTA.	40 MTA specimen Group 1: QMix Group2: 7% MA Group 3: 17% EDTA Group 4:distilled water	MA had a more detrimental effect on the physical properties of MTA, while EDTA was more harmful to the hydration of MTA.
Shekhar et al. 2022 [43] F1000Research	To use a CLSM to compare the effect of saline, EDTA, MA, and CA solutions on removal of smear layer and to measure depth of penetration of Bio ceramic sealer (BioRoot tmRCS) into the dentinal tubules at the coronal, middle and apical third regions of the root canal.	32 mandibular premolar teeth Group 1: saline, Group 2: 17% EDTA Group 3: 10% CA Group 4: 7% MA	The maximum penetration of bio-ceramic sealer was observed in the coronal region. At the apical third, the maximum sealer penetration was seen with 7% MA.
Anju et al. 2022 [44] Pesquisa Brasileira em Odontopediatria e Clínica Integrada	To evaluate and compare the effect of 17% EDTA, 9% HEDP, and 7% MA on the POBS of NeoMTA Plus sealer to the coronal, middle, and apical thirds of root canal dentin	40 single-rooted maxillary central incisors EDTA, HEDP, MA or Saline	MA and EDTA showed the highest POBS, with no significant difference between them. HEDP and saline had lower POBS, and the coronal third exhibited the highest values, followed by the middle and apical thirds.
Ballal et al. 2018 [45] Australian Dental Journal	To evaluate the effect of distilled water, EDTA, PA and MA on BD regarding surface topography, microhardness and POBS.	52 cylindrical shaped BD specimens Group 1: distilled water Group 2: 17% EDTA Group 3: 37% PA Group 4: 7% MA	EDTA, MA, and PA altered the surface morphology of BD. PA had microhardness similar to distilled water, while MA and EDTA showed reduced values compared to PA. PA also improved the POBS of BD compared to the control.
Ballal et al. 2019 [46] Saudi Endodontic Journal	To evaluate the effect of SmearOFF, 7% MA and 17% EDTA, on the surface microhardness of BD.	40 BD cylindrical-shaped specimens Group 1: 17% EDTA Group 2: 7% MA Group 3: SmearOFF solution Group 4: DW	MA significantly compromised the microhardness of BD, followed by EDTA, SmearOFF, and DW, with the differences being significant. When comparing 17% EDTA and SmearOFF, 17% EDTA caused the greatest reduction in microhardness.
Ballal et al. 2018 [47] Microscopy Research and Technique	To compare the effects of different chelating agents on the POBS of calcium silicate-based cements to the simulated root-end cavities.	50 maxillary anterior teeth Group 1: 17% EDTA Group 2: 7% MA Group 3: QMix Group 4: 2.25% PAA Group 5: 0.9% saline	Saline irrigation gave the highest bond strength in retrograde cavities with MTA or BD. EDTA and PAA showed better dislodgement resistance with BD. Irrigation choice impacts bond strength in root-end fillings.
Thakkar et al. 2021 [48] Pesquisa Brasileira em Odontopediatria e Clínica Integrada	To compare the effect of MA and Irritrol (combination of CHX & EDTA) irrigation on the sealing ability of BD when used as root-end filling material.	30 single-rooted human premolars Group 1: 7% MA Group 2: Irritrol Group 3: 0.9% Saline	Specimens irrigated with Irritrol showed the least microleakage compared to 7% MA and 0.9% Saline.
Ballal et al. 2013 [49] Journal of Dentistry	To evaluate the wettability of AH Plus and ThermaSeal Plus sealers on intraradicular dentine treated with different irrigating solutions.	50 anterior teeth Group 1: 2.5% NaOCl + QMix Group 2: 2.5% NaOCl + 17% EDTA Group 3: 2.5% NaOCl + 7% MA Group 4: 2.5% NaOCl Group 5: distilled water	QMix enhances the wettability of root canal dentin for both AH Plus and ThermaSeal Plus sealers. MA demonstrates promising results compared to EDTA and NaOCl. Dentin irrigated with EDTA shows the lowest wettability for both sealers.
Gandhi et al. 2020 [50] Saudi Endodontic Journal	To evaluate the effect of different chelating agents on the wettability of AH Plus and BioRoot RCS sealers on intraradicular dentin	50 single-rooted premolars Group 1: 2.5% NaOCl − 17% EDTA Group 2: 2.5% NaOCl-7% MA Group 3: 2.5% NaOCl-SmearOFF™ Group 4: 2.5% NaOCl-Dual Rinse^®^ HEDP Group 5: 2.5% NaOCl-DW	MA showed the best wettability on intraradicular dentin for both sealers. AH Plus had better wettability with SmearOFF™, followed by EDTA, Dual Rinse^®^ HEDP, and distilled water. BioRoot RCS showed better wettability with SmearOFF™ and EDTA, with SmearOFF™ being superior.
Ballal et al. 2012 [51] Australian Dental Journal	The efficacy of 10% CA, 17% EDTA and 7% MA with ultrasonic agitation in the removal of CH from root canals.	70 maxillary anterior teeth Group 1: CH + iodoform + silicone oil group 2: CH + PG Subgroups: CA, EDTA, MA	All irrigants completely removed CH + PG with no differences between them. MA and CA were significantly more effective than EDTA in removing CH + iodoform + silicone oil, with no difference between MA and CA.
Nainan et al. 2013 [52] Journal of Conservative Dentistry	To assess the efficacy of 17% EDTA and 7% MA in the removal of 3 CH preparations placed as intracanal medicaments.	60 single rooted premolars Group 1: Pure calcium hydroxide mixture with distilled water Group 2: ApexCal Group 3: Metapex; sub group 1: 17% EDTA, sub group 2: 7% MA	17% EDTA and 7% MA efficiently removed CH-distilled water mixture and Apexcal, with 7% MA showing better retrieval of Metapex than 17% EDTA.
Chandrashekhar et al. 2020 [53] Macedonian Journal of Medical Sciences	To evaluate the effect of various intermediate irrigating solutions in the removal of orange-brown precipitate formed due to alternative use of NaOCl and CHX root canal irrigants.	50 mandibular premolars Group 1: No intermediate irrigant Group 2: Saline, Group 3: 7% MA Group 4: 4% sodium thiosulfate Group 5: 70% isopropyl alcohol	Orange-brown precipitate (parachloroaniline) was observed in all groups except Group 4 and Group 5, which showed highly significant differences at all levels of the root canal.
Ballal et al. 2013 [54] The European Journal of Prosthodontics and Restorative Dentistry	To evaluate the effect of 7% MA and 17% EDTA on the shear bond strength of RealSeal SE sealer to root canal dentin	20 incisors Group 1: saline Group 2: 2.5% NaOcl followed by 7% MA Group 3: 2.5% NaOcl followed by 17% EDTA	No significant difference in bond strength was found between MA and 17% EDTA in the coronal and middle thirds. However, 7% MA showed higher bond strength in the apical third. The lowest bond strength was observed with saline, while the highest bond strength was in the apical third for both 7% MA and 17% EDTA.
Ballal et al. 2010 [55] Oral surgery, Oral Medicine, Oral Pathology, Oral Radiology, and Endodontics	To evaluate the postobturation apical seal following irrigation with 7% MA or 17% EDTA using dye leakage under vacuum method.	70 single-rooted human anterior teeth Group 1: 17% EDTA + 2.5% NaOCl Group 2: 7% MA + 2.5% NaOCl, Group 3: 0.9% saline	MA showed the least apical leakage, followed by EDTA, while saline exhibited the maximum leakage.
Ballal et al. 2013 [56] Clinical Oral Investigations	To evaluate and compare the genotoxic and apoptotic effect of aqueous solutions of eEDTA with that of MA using Chinese hamster lung fibroblast (V79) cells growing in vitro.	Exponentially growing V79 cells various concentrations of EDTA or MA alone	Both MA and EDTA are not considered genotoxic agents, with MA inducing less apoptotic and necrotic cell death compared to EDTA at clinically relevant doses.
Ballal et al. 2020 [57] Oral surgery, Oral Medicine, Oral Pathology, Oral Radiology, and Endodontics	To evaluate the antimicrobial efficacy of 7% MA and 17% EDTA in elimination of Enterococcus faecalis, Candida albicans, and Staphylococcus aureus at different time intervals.	Culture of bacterial strains *S. aureus* (ATCC 29,212) and *E. faecalis* (ATCC 29,212) and fungal strain *C. albican* 7% MA and 17% EDTA suspensions	No significant difference was found between MA and EDTA for all organisms at any time interval. However, significant differences were observed within the MA and EDTA groups at different time intervals.
Ballal et al. 2011 [58] International Endodontic Journal	To compare in vitro, the tissue-dissolution capacity of 7% MA, 17% EDTA, 2.5% NaOCl and 0.9% NaCl on human pulp tissue	40 pieces of human pulp tissue Group 1: 7% MA solution Group 2: 17% EDTA solution Group 3 : 2.5% NaOCl solution, Group 4: 0.9% NaCl solution	2.5% NaOCl dissolved pulp tissue significantly more than the other solutions. No significant difference in pulp dissolution was observed between 7% MA and 17% EDTA.
Ballal et al. 2011 [59] Oral surgery, Oral Medicine, Oral Pathology, Oral Radiology, and Endodontics	To evaluate mineral contents of root canal dentin after treatment with 7% MA or 17% EDTA.	30 pieces of teeth Group 1: 17% EDTA Group 2: 7% MA Group 3: saline	MA reduced the most calcium and phosphorus at all time intervals, but significantly only up to 5 min. Saline decreased oxygen, sulfur, and magnesium more than MA, while MA decreased sodium more than EDTA.
Khosla et al. 2017 [60] Indian Journal of Public Health Research & Development	To compare calcium loss and microhardness reduction of radicular dentin following treatment with 17% EDTA, 10% CA, 5% MA, and MTAD, by estimating calcium loss, and radicular dentin microhardness by using Atomic absorption spectrophotometry and by Vicker’s hardness tester	72 human mandibular premolars Group IA- 17% EDTA, Group IIA-10% CA Group IIIA- Biopure MTAD, Group IV A- 5% MA Group VA- Saline	All experimental chelating agents caused calcium loss and a reduction in microhardness of radicular dentin.
Ballal et al. 2011 [61] Journal of Endodontics	To evaluate the interaction between 7% MA and 2% CHX and to find out the availability of individual irrigant and to determine the free available chlorine content when 7% MA was mixed with 2.5% NaOCl solution	CHX standard solutions: 25, 50, 75, 100, 150 mg/mL from a 2% CHX solution. MA standard solutions: 50, 100, 150, 200, 250 mg/mL from a 7% MA solution. MA and CHX Solution	When combined with CHX, more than 90% of free MA and CHX were available, with no precipitate formation observed. However, the available chlorine content decreased significantly in the MA/NaOCl mixture.
Ballal et al. 2015 [62] The European Journal of Prosthodontics and Restorative Dentistry	To evaluate the effect of chlorine dioxide and various other more common irrigation solutions on the microhardness and surface roughness of root canal dentin	50 maxillary central incisors Group 1: 13.8% chlorine dioxide, Group 2: 17% EDTA Group 3: 7% MA Group 4: 2.5% NaOCl Group 5: Saline	Chlorine dioxide and NaOCl reduced microhardness more than other agents, while MA increased surface roughness. Chlorine dioxide should be used cautiously to prevent tooth damage.
Ballal et al. 2010 [63] Journal of Endodontics	To evaluate the effect of 7% MA and 17% EDTA solutions on the microhardness and the surface roughness of human root canal dentin	45 maxillary central incisors Group 1: 17% EDTA Group 2:7% MA Group 3:saline	There was no significant difference between EDTA and MA in reducing microhardness. However, MA significantly increased roughness more than EDTA.
Dave et al. 2023 [64] Journal of Conservative Dentistry and Endodontic	To evaluate the effectiveness of ultrasonic activation of EDTA, MA, and FA in combination with NaOCl on post endodontic treatment root fracture toughness.	40 single-rooted mandibular premolars Group 1: nonprepared Group 2 :Saline and NaOCl Group 3: 17% EDTA and NaOCl Group 4:, 7% MA and NaOCl Group 5: 0.7% FA and NaOCl	0.7% FA provided better root fracture resistance than both EDTA and MA.
Yadav et al. 2021 [65] Journal of Conservative Dentistry	To assess the effects of the three chelating agents as final rinse of the post space on bond strength of fiber posts luted with a self-adhesive resin cement.	45 mandibular premolars Group 1: 17% EDTA Group 2: 7% MA Group 3: 1% Phytic acid	Bond strength values improved with MA and phytic acid pre-treatment, with little to no significant difference between the groups. However, a final rinse with EDTA significantly reduced bond strength.
Basaiwala et al. 2017 [66] Indian Journal of Public Health Research and Development	To evaluate and compare the effect of RC-Prep, Canalizer, MA and CA on the microhardness of root canal dentine	20 single rooted maxillary and mandibular anterior human teeth MA and Citric acid	Microhardness decreased most with MA, followed by RC Prep and Canalizer. CA caused the least reduction in microhardness.
Ulusoy et al. 2024 [68] PloS One	To evaluate the dislodgement resistance of ProRoot MTA, Ortho-MTA, and Retro-MTA from the root canal dentine and its structural changes after exposure to 2.5% NaOCl, 2.5% NaOCl-17% EDTA, Dual Rinse HEDP, and 2.5% NaOCl-7% MA.	150 dentine slices Group1: ProRoot MTA Group2: Retro MTA, Group3: Ortho MTA. Subdivided into 2.5% NaOCl-17% EDTA, Dual Rinse HEDP, 2.5% NaOCl-7% MA, 2.5% NaOCl, distilled water	DR HEDP showed higher dislodgement resistance than 17% EDTA and 7% MA in Ortho MTA and Pro-Root MTA samples. In the Retro MTA group, both DR HEDP and 17% EDTA had higher dislodgement resistance than 7% MA. EDTA and MA-treated samples were more amorphous and granular, while DR HEDP-treated samples had a petal-like shape. Calcium levels were lower in EDTA and MA-treated samples compared to DR HEDP.
Buldur et al. 2019 [69] European Oral Research	To evaluate the effects of 17% EDTA, 7% MA, 10% CA on the POBS of ProRooT MTA and ERRM putty.	80 single-rooted human teeth Group 1: 17% EDTA Group 2: 7% MA Group 3: 10% CA Group 4: Positive Control subgroups: Group A (ProRoot MTA) and Group B: ERRM putty	Both chelating agents and calcium silicate-based cement were significantly associated with POBS values. The bond strength was significantly lower for CA compared to EDTA or MA. ERRM exhibited higher bond strength values than ProRoot MTA.
Gokturk et al. 2020 [70] Journal of Conservative Dentistry	To evaluate the impact of 7% MA, 17% EDTA solution, 10% CA, or 2.25% PAA on the dislodgment resistance of a silicone-based root canal sealer.	95 mandibular incisors Group 1: 5% NaOCl Group 2: 17% EDTA Group 3: 7% MA, Group 4: 10% CA, Group 5: 2.25% PAA	Roots irrigated with chelators had significantly higher bond strength than those irrigated with NaOCl. MA showed the highest bond strength, with no significant difference between the other chelating solutions.
Arslan et al. 2014 [71] Journal of Endodontics	To evaluate the effect of various irrigating solutions on the removal of CH mixed with 2% CHX gel from an artificial groove created in a root canal and the generation of orange-brown precipitate in the remaining CH mixed with 2% CHX gel after irrigation with the various irrigating solutions	48 mandibular premolars Group 1: 1% NaOCl Group 2: 17% EDTA Group 3: 7% MA Group 4: 10% CA	7% MA and 10% CA outperformed 1% NaOCl and 17% EDTA in removing calcium hydroxide with 2% CHX gel. No differences were seen in the other groups. Orange-brown precipitate was only observed in the NaOCl group.
Alim et al. 2021 [72] Microscopy Research and Technique	To investigate the effects of different chelation solutions on the penetration of resin-based and bioceramic root canal sealers into dentinal tubules using a device that assembles conventional microplate detection with automated digital microscopy	84 single-rooted teeth Group 1:saline Group 2: EDTA Group 3: MA Group 4: HEBP	MA group had the deepest penetration in the apical third. In the middle and coronal thirds, EDTA and HEBP showed similar depths. Penetration was highest in the coronal thirds across all irrigation groups. EndoSequence BC Sealer outperformed AH Plus and MTA Fillapex, but differences were not significant. Chelation solutions improved sealer penetration into dentinal tubules.
Kara et al. 2012 [73] Journal of Endodontics	To evaluate the effects of different solutions used for final irrigation on sealer penetration into dentinal tubules	32 mandibular premolar teeth Group 1: 17% EDTA + 2.5% NaOCl Group 2: 7% MA + 2.5% NaOCl Group 3:10% CA + 2.5% NaOCl Group 4: 2.5% NaOCl	Final irrigation with EDTA, MA, and CA after NaOCl use affected sealer penetration, but no significant differences were found between these groups in any section.
Ozasir et al. 2021 [74] Scanning	The effects of different final irrigation regimens on the dentin tubule penetration of three different root canal sealers using CLSM.	160 single-rooted mandibular premolar teeth Group 1: 7% EDTA Group 2: 17% EDTA & 2% CHX Group 3: 7% MA Group 4: 7% MA & 2% CHX group 5: 5.25% NaOCl	Tech BioSealer Endo showed least dentinal tubule penetration, while AH Plus performed better than EndoRez. Final irrigation with 7% MA or 17% EDTA showed no difference in sealer penetration. However, using 2% CHX after chelating agents improved penetration depth and percentage.
Ulusoy et al. 2014 [75] Brazilian Oral Research	To compare the effects of EDTA and MA on the sealing ability of various root canal sealers	80 root canals Group 1: MA + Hybrid Root SEAL/GP. Group 2: EDTA + Hybrid Root SEAL/ Group 3: MA + iRoot SP/GP Group 4: EDTA + iRoot SP/GP Group 5: MA + EndoREZ/EndoREZ points Group 6: EDTA + EndoREZ/EndoREZ points Group 7: MA + AH Plus/GP Group 8: EDTA + AH	AH Plus and EndoREZ had the lowest microleakage, while Hybrid Root SEAL had the highest. MA caused higher microleakage than EDTA. Final irrigation solution influences the apical seal, with AH Plus and EndoREZ providing better sealing than IRoot SP and Hybrid Root SEAL.
Ulusoy et al. 2014 [76] PloS One	the dislodgement resistance and structural changes of different MTA like Pro-Root MTA, Ortho MTA, and Retro MTA after exposure to NaOC, NaOCl-EDTA, Dual Rinse HEDP and NaOCl- MA.	150 dentine slices Group1: ProRoot MTA, Group2: Retro MTA, and Group3: Ortho MTA.	Calcium level was decreased more in samples treated with 17% EDTA and 7% MA when compared to DR HEDP.
Kara et al. 2015 [77] Australian Dental Journal	To evaluate the effects of QMix, EDTA + CHX, EDTA + NaOCl and MA on the microhardness of root canal dentine.	40 extracted human maxillary canine teeth Group 1: 17% EDTA + 2.5% NaOCl Group 2: 17% EDTA + 2% CHX Group 3: QMix Group 4: 7% MA	MA significantly reduced microhardness in all regions compared to other groups. In the coronal and middle regions, no significant differences were found among the groups. In the apical region, QMix and 17% EDTA + 2% CHX showed no significant difference, but both caused greater microhardness reduction than 17% EDTA + 2.5% NaOCl.
Gokturk et al. 2019 [78] Journal of Oral Science	To evaluate the degree of crack formation during canal preparation using reciprocating files	120 incisor teeth Group 1:distilled water Group 2:NaOCl Group 3:CA Group 4:MA Group 5: PAA Group 6: EDTA gel Group 7:EDTA liquid	The chelating agents produced similar numbers of cracked sections. Using a chelating agent during preparation with reciprocating instruments helped reduce crack formation.
Topbaş et al. 2022 [79] BMC Oral Health	To investigate the effects of different root canal irrigation protocols applied to the dentin and artificial aging procedures on the mPBS between dentin and hybrid ceramic posts.	75 single-rooted mandibular premolar teeth Group 1:SS Group 2: NaOCl + SS Group 3:EDTA + NaOCl + SS Group 4: MA + NaOCl + SS Group 5:Ch + NaOCl + SS	MA + NaOCl + SS had the highest mPBS. The coronal zone had the highest mPBS, and aging reduced it significantly. Cohesive and mixed failures were most common. 7% MA (Group 4) was more effective than 17% EDTA (Group 3) at removing the smear layer, particularly in the apical zone.
Giardino et al. 2020 [80] International Endodontic Journal	To evaluate the antimicrobial, toxicity and EDTA and MA alone and combined with CTR	Chinese hamster cells V79 Group 1: 17% EDTA Group 2: 17% EDTA + 0.5% CTR Group 3: 7% MA Group 4: 7% MA + 0.5% CTR	7% MA was less cytotoxic than 17% EDTA. Adding CTR to both EDTA and MA enhanced debris removal from canal walls and boosted their antimicrobial activity compared to solutions without detergents.
Rifaat et al. 2023 [81] European Journal of Dentistry	To assess the bonding strength of EndoSeal MTA and AH Plus sealers after using three irrigation protocols as follows: 17% EDTA, 7% MA, and 37% PA.	60 middle root slices of 1-mm thickness each Group 1: 17% EDTA, Group 2: 7% MA Group 3: 37% PA	The irrigation protocol significantly affected the bond strength of EndoSeal MTA and AH Plus sealers. AH Plus showed the highest bond strength with 7% MA, followed by 37% PA 17% EDTA. In contrast, EndoSeal MTA exhibited the highest bond strength with 17% EDTA, followed by 7% MA and 37% PA.
Basmaci et al. 2013 [82] International Endodontic Journal	To evaluate ex vivo the effectiveness of single-file instrumentation techniques compared with serial Ni-Ti rotary instrumentation with several irrigation regimens in reducing E. faecalis within root canals.	81 extracted human mandibular premolar teeth Group 1-A: phosphate-buffered saline + SAF, group 1-B: 5% NaOCl + 15% EDTA + SAF, group 1-C: 5% NaOcl + 7% MA + SAF, group 2-A: phosphate-buffered saline + Reciproc (R25), group 2-B: 5% NaOCl + 15% EDTA + Reciproc (R25), group 2-C: 5% NaOCl + 7% MA + Reciproc (R25), group 3-A: phosphate-buffered saline + ProTaper, group 3-B: 5% s NaOcl + 15% EDTA + ProTaper, group 3-C: 5% NaOCl + 7% MA + ProTaper	All techniques and irrigation regimens significantly reduced the number of bacterial cells in the root canal
Ordinola-Zapata et al. 2013 [83] Acta Odontologica Scandinavica	To evaluate if the incorporation of antimicrobial compounds to chelating agents or the use of chelating agents with antimicrobial activity as 7% MA and PAA show similar disinfection ability in comparison to conventional irrigants as NaOCl or iodine potassium iodide against biofilms developed on dentin.	Bio-volume of live cells Group 1: MTAD Group 2: Qmix Group 3:Smear Clear Group 4:7% MA Group 5: 2% iodine potassium iodide Group 6: 4% PAA Group 7: 2.5% & 5.25% NaOCl	Irrigants like QMix, Smear Clear, MA, iodine compounds, and MTAD were ineffective against biofilm on infected dentin. However, 4% PAA and 2.5–5.25% NaOCl significantly reduced bacteria and cleaned dentin surfaces.
Wang et al. 2017 [84] Acta Odontologica Scandinavica	To evaluate the effect of MA on the cleaning efficacy and mechanical properties of root canal dentine with respect to different time exposure.	80 single-canal premolars Group 1: 7% MA + 2.5% NaOCl Group 2: 17% EDTA + 2.5% NaOCl Group 3: QMix + 2.5% NaOCl Group 4: 0.9% saline	The cleaning efficacy and mechanical properties of root canal dentine depended on MA exposure time. A 45-second application was most suitable for clinical use, enhancing tooth fracture resistance. 1 min treatment caused irreversible dentine erosion and reduced mechanical properties, 3 min treatment resulted in the lowest fracture resistance.
Fan et al. 2017 [85] European Journal of Oral Sciences	To evaluate the effect of MA on both the bond strength of fibre post to root dentine and smear layer removal after post space preparation	60 single-canal premolars Group 1: 0.9% saline Group 2: 2.5% NaOCl Group 3: 17% EDTA followed by 2.5% NaOCl Group 4: 7% MA followed by 2.5% NaOCl	MA had the highest bond strength in the apical region and was most effective at smear layer removal, especially in the apical area. It performed better than other groups and is an efficient final irrigant for opening dentinal tubules and removing the smear layer.
Baca et al. 2011 [86] Journal of Endodontics	To evaluate the residual antimicrobial activity of four final irrigation regimens in root canals contaminated with Enterococcus faecalis.	Biofilms of E. faecalis grown in uniradicular roots Group 1: 17% EDTA-NaOCl Group 2: 7% MA-NaOCl Group 3: 17% EDTA-2% CHX + 0.2% CTR, Group 4: 7% MA- 2% CHX + 0.2% CTR	The combination of 2% CHX + 0.2% CTR would be an effective alternative final irrigation regimen given its antimicrobial action over time.
Baca et al. 2011 [87] Journal of Endodontics	To evaluate the residual antimicrobial activity and the capacity to eradicate Enterococcus faecalis biofilm of different irrigating solutions, alone and in combination, in a dentin-volumetric test.	Treated dentin blocks to E. faecalis Group 1: 2.5% NaOCl Group 2: 2% CHX Group 3: 0.2% CTR Group 4: 17% EDTA Group 5: 7% MA and regimens of 2.5% NaOCl followed by 17% EDTA or 7% MA and 0.2% CTR or 2% CHX	A 2% CHX and 0.2% CTR solution inhibited 100% of the biofilm, while 2.5% NaOCl showed the lowest residual activity. The kill percentage was highest with 2.5% NaOCl and 0.2% CTR, followed by 7% MA and 2% CHX. Solutions of 7% MA or 17% EDTA followed by 0.2% CTR or 2% CHX showed 100% residual and antimicrobial activity
Ferrer-Luque et al. 2012 [88] Journal of Endodontics	To evaluate the residual antimicrobial activity of 17% EDTA, 7% MA, and 10% LA alone and combined with 0.2% CTR on infected root canals with Enterococcus faecalis.	Biofilms of E. faecalis grown in uniradicular roots Group 1: 17% EDTA; Group 2: 17% EDTA + 0.2% CTR Group 3: 7% MA Group 4: 7% MA + 0.2% CTR Group 5: 10% LA Group 6: 10% LA + 0.2% CTR	The residual activity against E. faecalis of 7% MA + 0.2% CTR, 17% EDTA + 0.2% CTR, and 10% LA + 0.2% CTR was greater than the use of chelating agents alone.
Ferrer-Luque et al. 2010 [89] Journal of Endodontics	To evaluate the in vitro capacity of MA in eradicating Enterococcus faecalis biofilms and to evaluate the combinations of CTR with MA, CA, and EDTA	E. faecalis biofilms dilutions of MA (7%−0.01%),combinations of 0.2% CTR with 7% MA, 15% EDTA, and 15% CA	MA eradicated E. faecalis biofilms at 0.88% after 30 s and at 0.11% after 2 min. When combined with 0.2% CTR, it eliminated the biofilms at all exposure times.
Ferrer-Luque, et al. 2015 [90] Journal of Biological Research-Thessaloniki	To determine the residual antimicrobial activity of several final irrigation protocols with 7% MA alone and combined with CHX, CTR or both, in root canals infected with Enterococcus faecalis.	Biofilms of E. faecalis Group 1: 2.5% NaOCl Group 2: 7% MA Group 3: 7% MA + 0.2% CTR Group 4: 7% MA + 2% CHX Group 5: 7% MA + 0.2% CTR + 2% CHX; Group 6: 0.9% saline	The combination of MA with CHX and CHX + CTR as final irrigants showed the best results, with no significant differences between them, followed by 7% MA + 0.2% CTR. The 7% MA group showed significant differences compared to the groups where MA was combined with CHX and CHX + CTR.
Ferrer-Luque et al. 2013 [90] Medicina Oral, Patología Oral, Cirugía Bucal	To determine the decalcifying efficacy of 7% MA, 2% CHX, and combinations of 7% MA + 0.2% CTR and 2% CHX + 0.2% CTR, in four time periods	40 sections Group 1:7% MA Group 2:2% CHX Group 3: 7% MA + 0.2% CTR Group 4: 2% CHX + 0.2% CTR	Significant differences were observed in calcium extraction across all time periods. 7% MA extracted the most calcium at all immersion times, followed by 7% MA + 0.2% CTR. 2% CHX and its combination with 0.2% CTR extracted almost no calcium.

*MA* maleic acid, *EDTA* ethylenediaminetetraacetic acid, *NaOCl* sodium hypochlorite, *HEPD* etidronic acid, *PAA* peracetic acid, *CHX* chlorhexidine, *CA* citric acid, *MTA* mineral trioxide aggregate, *CLSM* confocal laser scanning microscopy, *CTR* cetrimide, *GP* gutta-percha, *LAL* laser activated irrigation, *BD* biodentine, *PG* propylene glycol, *PA* phosphoric acid, *POBS* push-out bond strength, *FA* fumaric acid, *ERRM* endosequence root repair material, *Nacl* saline, *SS* saline solution, *LA* lactic acid, *mPBS* micro pushout bond strength, *CH* calcium hydroxide, *SAF* Self adjusting file, *DW* distilled water, *MTAD* Mixture of Tetracycline, Acid, Detergent, Dual Rinse HEDP: 1-hydroxyethylidene-1, 1-bisphosphonate

### Smear layer removal

A number of studies have assessed smear layer removal as a parameter from various sections of the root canal, utilising different irrigants and comparing their effectiveness to that of 7% MA [13, 15, 27–39, 67]. The findings varied among the studies. In the apical third, most studies indicated that 7% MA was more effective than various other irrigants, including 17% EDTA, 18% etidronic acid, 5% glycollic acid, 0.5% peracetic acid (PAA), 10% CA, SmearOFF, and SmearClear. Gupta et al. [33] demonstrated that the Mixture of Tetracycline, Acid, Detergent (MTAD) exhibited comparable efficacy to 10% MA in the apical third.

Attur et al. [13] found that 17% EDTA was more effective than 7% MA and 2% CHX across all thirds of the root canal. Ballal et al. [28] observed no significant difference between 17% EDTA and 7% MA in the coronal and middle thirds. Both methods demonstrated comparable efficacy in the elimination of the smear layer, however in the apical third, 7% MA demonstrated a significantly superior ability to remove the smear layer compared to 17% EDTA. Saju et al. [30] found that 0.2% chitosan nanoparticles were more effective in removing smear layers compared to 17% EDTA and 7% MA when using the XP Endo finisher. Butala et al. [34] demonstrated that 7% MA exhibited greater efficacy compared to 17% EDTA and 0.5% PAA in the middle third of the root canal. Kaushal et al. [35] found that the smear layer from the coronal and middle thirds was effectively removed by both 7% MA and 10% CA. The effectiveness of a diode laser combined with 7% MA and 17% EDTA in removing smear layers was investigated by Nabi et al. [36], who concluded that the diode laser with 7% MA outperformed the diode laser with 17% EDTA. Meshram et al. [37] found that in the middle third, 10% Dimercaptosuccinic acid and 2.5% NaOCl demonstrated greater efficacy compared to 7% MA and 17% EDTA. Rao et al. [38] found no significant difference between SmearOff and 7% MA in the coronal, middle, and apical thirds; however, 17% EDTA demonstrated reduced effectiveness in the apical third. Varghese et al. [39] investigated the effect of 7% MA at time intervals of 1 and 3 min, concluding that both intervals effectively removed the smear layer.

### Additional effects

Multiple in-vitro studies have investigated the effects of MA on various parameters pertinent to endodontic therapy.

#### Effect on growth factors release

To date, only one in vitro investigation has assessed the release of TGF-β1 with the application of 7% MA. Ballal et al. [40] established that 7% MA in conjunction with 1.5% NaOCl generated a greater quantity of growth factors compared to 17% EDTA with 1.5% NaOCl in both biofilm and no biofilm conditions. In nonbiofilm samples, the release of growth factors was much greater with 7% MA alone compared to 17% EDTA.

#### Effects on dental materials

A total of 28 in vitro investigations assessed the impact of 7% MA on several dental materials. Shekhar et al. [43] and Alim et al. [72] determined that the greatest sealer penetration in the apical third occurred with 7% MA. Kara et al. [73] and Ozasir et al. [74] saw no significant difference in sealer penetration between 7% MA and 17% EDTA, however 2% CHX enhanced total depth.

Anju et al. [44] and Rifaat et al. [80] discovered that 7% MA enhanced push-out bond strength (POBS) for Neo mineral trioxide aggregate Plus and AH Plus sealers, particularly in the coronal and apical thirds, respectively. Buldur et al. [69] observed that 7% MA and 17% EDTA enhanced bond strength using calcium silicate-based cements more successfully than 10% CA. Gokturk et al. [70] indicated that 7% MA attained the maximum binding strength using silicone-based sealers, surpassing 2.5% NaOCl and 17% EDTA. Ballal et al. [54] discovered that 7% MA enhanced the shear bond strength of Reaseal SE sealer in the apical third.

Ballal et al. [45] indicated that 7% MA modified Biodentine’s surface shape, whereas 37% phosphoric acid (PA) enhanced its post-operative bond strength. Ballal et al. [46] observed that 7% MA adversely affected the physical characteristics and microhardness of both mineral trioxide aggregate (MTA) and Biodentine to a greater extent than 17% EDTA and SmearOFF. Ulusoy et al. [75] discovered that 7% MA resulted in more microleakage compared to 17% EDTA. Thakkar et al. [48] discovered that 7% MA resulted in increased microleakage relative to Irritrol.

Ballal et al. [51] determined that 7% MA and 10% CA were more effective than 17% EDTA and 2.5% NaOCl in the elimination of calcium hydroxide. Arslan et al. [71] concluded that 7% MA and 10% CA were more effective than 1% NaOCl and 17% EDTA in the elimination of calcium hydroxide. Nainan et al. [52] indicated that 7% MA was more efficacious than 17% EDTA in the removal of Metapex. Pallepagu et al. [20] discovered that 10% MA was more effective than 17% EDTA in eliminating Odontopaste. Chandrashekhar et al. [53] noted that 7% MA was less efficacious than sodium thiosulfate and isopropyl alcohol in eliminating the 2.5% NaOCl-CHX precipitate.

Ulusoy et al. [68] discovered that Dual Rinse 1-hydroxyethylidene-1, 1-bisphosphonate (DR HEDP) had greater dislodgement resistance than both 17% EDTA and 7% MA. Ballal et al. [46] discovered that 17% EDTA and 0.5% PAA exhibited superior dislodgement resistance with Biodentine compared to 7% MA and QMix.

Ballal as al. [49] and Gandhi et al. [50] discovered that 7% MA enhanced the wettability of AH Plus and BioRoot RCS sealers; nevertheless, AH Plus exhibited superior wettability with SmearOFF™, succeeded by EDTA, DR HEDP, and distilled water. BioRoot RCS exhibited enhanced wettability with SmearOFF and EDTA, with SmearOFF demonstrating superior performance.

Ballal et al. [41] indicated that 7% MA and DR HEDP exhibited more efficacy than 17% EDTA in the removal of MTA Fillapex. Ballal et al. [42, 46] similarly determined that 7% MA adversely impacted the physical qualities of MTA to a greater extent. Ballal et al. [55] noted that 7% MA exhibited the minimal apical leakage in comparison to alternative irrigants.

#### Effect on antimicrobial afficacy

Eleven studies assessed the antimicrobial effects of 7% MA. Ballal et al. [56] demonstrated that 7% MA resulted in reduced apoptotic and necrotic cell death, exhibiting lower toxicity at clinically relevant concentrations. Ballal et al. [57] and Basmaci et al. [81] reported that 7% MA and 17% EDTA significantly decreased bacterial counts, although the effects varied over time. Ballal et al. [58] compared the pulp dissolution capacities, revealing that 7% MA and 17% EDTA exhibited similar performance, yet both were less effective than 2.5% NaOCl, while 0.9% saline (NaCl) showed no effect.

Giardino et al. [79] observed that 7% MA exhibited lower cytotoxicity compared to 17% EDTA. Additionally, the incorporation of cetrimide (CTR) into both 7% MA and 17% EDTA enhanced their antimicrobial efficacy. Ordinola-Zapata et al. [82] observed that 7% MA combinations with QMix, Smear Clear, and iodine compounds were ineffective against biofilm, whereas 4% PAA and 2.5–5.25% NaOCl demonstrated greater efficacy.

Baca et al. [85]– [86] found that 7% MA in combination with NaOCl or 2% CHX + 0.2% CTR demonstrated lower efficacy compared to 2% CHX + 0.2% CTR alone. Ferrer-Luque et al. [87] demonstrated that a combination of 7% MA and 0.2% CTR exhibited greater residual antimicrobial activity compared to 7% MA or 17% EDTA used individually. Furthermore, E. faecalis biofilms were eradicated by 7% MA alone at concentrations of 0.88% after 30 s and 0.11% after 2 min. When combined with 0.2% CTR, the biofilms were effectively eliminated at all exposure times, indicating improved biofilm eradication potential [88]. The study concluded that the combination of 7% MA with 2% CHX and 2% CHX + 0.2% CTR as final irrigants demonstrated superior antimicrobial activity, with the 7% MA group exhibiting better results compared to the other combinations [89].

#### Effect on inorganic mineral contents of dentin

Numerous research evaluated the effect of 7% MA on dentin mineral composition. Ballal et al. [59] discovered that 7% MA and QMix considerably diminished phosphorus and magnesium levels, although QMix resulted in a more substantial loss of calcium. Khosla et al. [60] noted that 5% MA, 17% EDTA, 10% CA, and Biopure MTAD all lowered calcium levels. Ulusoy et al. [91] observed reduced calcium levels in samples treated with 17% EDTA and 7% MA in comparison to DR HEDP. Ferrer-Luque et al. [90] established that 7% MA was the most efficacious in calcium extraction, whereas 7% MA combined with 0.2% CTR ranked second.

#### Effect on physical and mechanical properties of root Canal dentin

Eight studies evaluated the effect of 7% MA on dentin microhardness, roughness, and fracture resistance. Gupta et al. [33] discovered that MTAD and 10% MA diminished microhardness more significantly than 17% EDTA and 7% MA. Ballal et al. [62] indicated that 7% MA and 17% EDTA diminished microhardness, while 2.5% NaOCl resulted in much more significant reductions. Moreover, 7% MA enhanced surface roughness to a greater extent than 17% EDTA [62]– [63]. Dave et al. [64] determined that 7% MA and 17% EDTA diminished root fracture resistance to a lesser extent than 0.7% fumaric acid. Ulusoy et al. [67] noted that 5 min of 7% MA resulted in the most significant reduction in microhardness. Kara et al. [76] and Khosla et al. [60] confirmed that 7% MA, 17% EDTA, 10% CA, and MTAD decreased microhardness. Gokturk et al. [77] discovered that 7% MA decreased crack formation when utilising reciprocating instruments. Wang et al. [83] observed that a 45-second exposure to 7% MA improved fracture resistance, whereas extended exposure (3 min) resulted in erosion and diminished mechanical characteristics.

#### Effect on bond strength of post to root dentin

Three studies assessed the impact of fiber post on the binding strength to radicular dentin. Yadav et al. [65] discovered that bond strength values improved with 7% MA and 1% phytic acid preconditioning when fiber posts are luted using a self-adhesive resin cement. Topbaş et al. [78] established the highest micro-POBS between dentin and hybrid ceramic posts when 7% MA was utilised in conjunction with 2.5% NaOCl. Fan et al. [84] determined that 7% MA exhibited the strongest bond strength in the apical region when compared to 2.5% NaOCl and 17% EDTA.

## Discussion

A scoping review was selected because there is varied evidence on MA as a root canal irrigant, with research varying in their effects and outcomes. A systematic review with meta-analysis was impracticable due to this unpredictability and the restricted number of clinical research.

Numerous studies indicate that 7% MA is extremely efficacious in eliminating the smear layer, particularly in the apical third [15, 27–39, 67]. This phenomenon is ascribed to its low pH (≈ 1.03), which promotes demineralisation, while 17% EDTA functions predominantly via chelation. The apical region frequently exhibits sclerotic dentin and reduced concentrations of non-collagenous proteins (NCPs), potentially attenuating the chelating efficacy of 17% EDTA [27, 38]. Conversely, 7% MA continues to demonstrate efficacy under these circumstances. Nevertheless, research by Attur et al. [13] indicated that 17% EDTA is superior, probably owing to variations in methodology and testing conditions.

A potential explanation for the conflicting findings across various studies can be the variability in irrigant concentration and pH levels. MA has been evaluated at concentrations of 5%, 7%, and 10%, with increased concentrations resulting in enhanced smear layer removal, but accompanied by greater microhardness loss [28, 33].

In several investigations, MTAD shown comparable efficacy to 7% MA and exceeded EDTA, likely attributable to its capacity to generate a denser demineralised layer, decelerate collagen degradation, and sustain extended MMP-inhibitory action [33]. However, unlike MTAD, MA offers the advantage of lower cytotoxicity, simpler composition, and reduced cost, making it a promising alternative for both conventional and regenerative endodontic procedures.

The timing and quantity of application are also critical factors. Wang et al. [83] discovered that a 45-second exposure to 7% MA enhanced fracture resistance, suggesting that limited contact effectively removes the smear layer and conditions the dentin surface without causing structural compromise. In contrast, extending the application to three minutes resulted in pronounced dentin erosion and degradation of mechanical properties, indicating that overexposure may lead to excessive demineralisation. Ulusoy et al. [67] similarly indicated that extended exposure resulted in the most significant decrease in dentin microhardness. These findings highlight the delicate balance between achieving adequate smear layer removal and preserving dentin integrity.

Besides eliminating the smear layer, 7% MA improves adhesion. Research indicates that 7% MA markedly enhances cement infiltration into dentinal tubules, resulting in an increased formation of resin tags and elevated bond strength with fibre posts [44]. It enhances the adherence of calcium silicate-based cements and AH Plus sealer, likely due to its reduced surface tension [69]. Additionally, the formation of mineral gradients and the augmentation of surface roughness by7% MA improve sealer penetration and push-out bond strength [63]. Methodological discrepancies further exacerbate variability. The majority of investigations were performed in vitro or ex vivo, potentially failing to effectively represent clinical settings, including buffering effects or dynamic fluid exchange.

The order and interplay of irrigants are also essential as the interaction of MA with other irrigants significantly influences its clinical performance. When employed in sequence with NaOCl, MA does not induce rapid chlorine depletion as seen with the EDTA–NaOCl combination, thereby maintaining NaOCl’s tissue-dissolving efficacy and antibacterial properties; thus, 2.5% NaOCl outperformed 7% MA and 17% EDTA in pulp dissolution effectiveness [58]. Furthermore, the incorporation of CTR or CHX with MA has been shown to significantly enhance its antimicrobial and antibiofilm activity without adversely affecting dentin structure [85, 86, 88, 90], but its combination with QMix or iodine compounds proved ineffective against biofilms [82]. Furthermore, its demineralising effect facilitates the release of growth factors, including TGF-β1, which may aid in healing and regeneration [40]. However, this is based on a single study; hence, it cannot be regarded as convincing evidence.

These discrepancies elucidate why several research advocated using 7% MA, whereas others deemed it equivalent or inferior to EDTA or alternative irrigants. Despite variability, 7% MA consistently exhibited advantages in smear layer elimination, sealer infiltration, and bond strength, attributable to its low surface tension and capacity to alter dentin surface characteristics [44, 63, 69]. Despite its benefits, the acidic properties of 7% MA continue to pose a constraint, as they might diminish dentin microhardness and mineral concentration [34]. This dual effect, advantageous for growth factor release yet detrimental to structural integrity, underscores the necessity of optimising irrigation techniques to reconcile efficacy with the preservation of dentin integrity.

### Strengths and limitations

The application of 7% MA as a root canal irrigant is consistent with the United Nations Sustainable Development Goal 3: Good Health and Well-being. Its effective smear layer removal and antimicrobial properties contribute to reduced infection risks, decreased necessity for extractions and prosthetic replacements, and enhanced long-term oral health outcomes. This review presents specific limitations. Stakeholder consultation was absent for validating findings, obtaining additional insights, and improving the applicability of results. Furthermore, most of the included studies were in vitro, which restricts their direct relevance to clinical settings. Furthermore, the majority of studies concentrated on short-term outcomes, resulting in insufficient data regarding the long-term effects of 7% MA on dentin integrity. Additional in-vivo studies are necessary to determine the long-term efficacy and safety in endodontic practice.

## Conclusions

In conclusion, 7% MA exhibits reliable advantages as a root canal irrigant, especially in the apical area, due to its enhanced capacity to remove the smear layer, facilitate sealer penetration, and strengthen bond integrity. The ability to enhance surface roughness and facilitate adhesion underscores its significance as a viable alternative to conventional chelating agents in endodontics. The potential decrease in dentin microhardness highlights the necessity of balancing treatment efficacy with the preservation of dentin integrity. Variability among studies can be ascribed to variations in irrigant concentration, pH, duration of application, type of dentin, methodology, interactions of irrigants, outcomes evaluated, and characteristics of the sample. Future well-designed in vivo studies with standardised protocols are essential to establish the true clinical significance and ensure safe, predictable use.

## Supplementary Information

Supplementary Material 1

## Data Availability

The Appendix contains all of the study’s data and materials.

## Abbreviations

RCT: Root canal treatment
MA: Maleic acid
EDTA: Ethylenediaminetetraacetic acid
NaOCl: Sodium hypochlorite
CNP: Chitosan napoparticles
GA: Glycolic acid
PAA: Peracetic acid
CHX: Chlorhexidine
CA: Citric acid
SEM: Scanning electron microscope
DMSA: Dimercaptosuccinic acid
HEPD: Etidronic acid
MTA: Mineral trioxide aggregate
CLSM: Confocal laser scanning microscopy
CTR: Cetrimide
GP: Gutta-percha
LAL: Laser activated irrigation
BD: Biodentine
PG: Propylene glycol
PA: Phosphoric acid
POBS: Push-out bond strength
FA: Fumaric acid
ERRM: Endosequence root repair material
Nacl: Saline
SS: Saline solution
LA: Lactic acid
mPBS: Micro pushout bond strength
CH: Calcium hydroxide
SAF: Self adjusting file
DW: Distilled water
MTAD: Mixture of tetracycline, acid and detergent
DR HEDP: Dual rinse 1-hydroxyethylidene-1, 1-bisphosphonate
