# Supplementary material for: Maleic acid as a root canal irrigant- a scoping review
Source: BMC Oral Health. 2025 Dec 8;26:76. doi: 10.1186/s12903-025-07361-9 (PMC12801934; doi:10.1186/s12903-025-07361-9)
Supplement: Supplementary file 1 — Supplementary Material 1 [file 12903_2025_7361_MOESM1_ESM.docx]

| DATABASE | SEARCH STRATEGY | RESULTS / NO.OF HITS |
| --- | --- | --- |
| PUBMED(MEDLINE) | "maleic acid"[All Fields] OR maleic acid [Text Word] AND "root canal irrigants"[MeSH Terms] OR "root canal irrigants"[MeSH Terms] OR Root Canal Irrigant[Text Word] "chelating agents"[MeSH Terms] OR “chelating agents”[Text Word] OR "smear layer"[MeSH Terms] OR smear layer[Text Word] OR "endodontics"[MeSH Terms] OR endodontics[Text Word] OR root canal [Text Word] OR dental[Text Word]  Filters: English and medline | 142 |
| SCOPUS | ALL ( "maleic acid" ) OR TITLE-ABS-KEY ( "maleic acid" ) INDEXTERMS ( "root canal irrigants" ) OR INDEXTERMS ( "root canal irrigants" ) OR INDEXTERMS ( "Root Canal Irrigant " chelating AND agents "" ) OR TITLE-ABS-KEY ( "chelating agents" ) OR INDEXTERMS ( "smear layer" ) OR TITLE-ABS-KEY ( "smear layer" ) OR INDEXTERMS ( endodontics ) OR TITLE-ABS-KEY ( endodontics ) OR TITLE-ABS-KEY ( "root canal" ) OR TITLE-ABS-KEY ( dental ) AND ( LIMIT-TO ( SUBJAREA , "DENT" ) ) AND ( LIMIT-TO ( DOCTYPE , "ar" ) ) AND ( LIMIT-TO ( LANGUAGE , "English" ) ) | 837 |
| EMBASE | 'maleic acid'/exp OR 'maleic acid' AND 'biomedical and dental materials'/exp OR 'root canal irrigant' OR 'chelating agent'/exp OR 'chelating agents' OR 'dental surgery'/exp OR 'smear layer' OR 'endodontics'/exp OR 'endodontics' OR 'root canal' OR 'dental' AND [english]/lim AND [humans]/lim AND 'Article'/it AND 'dental' AND 'Article'/it | 453 |
| WEB OF SCIENCE | ALL="maleic acid" OR ALL="maleic acid" AND ALL=("Root Canal Irrigant" OR "chelating agents" OR "smear layer" OR "endodontics" OR "root canal" OR "dental") | 144 |

APPENDIX 1
